# MicroRNA miR-466 inhibits Lymphangiogenesis by targeting prospero-related homeobox 1 in the alkali burn corneal injury model

**DOI:** 10.1186/s12929-014-0104-0

**Published:** 2015-01-02

**Authors:** Minkoo Seo, Jun-Sub Choi, Chang Rae Rho, Choun-Ki Joo, Suk Kyeong Lee

**Affiliations:** Department of Medical Lifescience, College of Medicine, The Catholic University of Korea, Seoul, Korea; Catholic Institute for Visual Science, College of Medicine, The Catholic University of Korea, Seoul, Korea; Department of Ophthalmology and Visual Science, Daejeon St. Mary’s Hospital, Daejeon, Korea; Department of Ophthalmology and Visual Science, Seoul St. Mary’s Hospital, Seoul, Korea

**Keywords:** MicroRNA, Prox1, miR-466, miR-181, Tube Formation, Lymphangiogenesis, Cornea transplantation, Alkali burn

## Abstract

**Background:**

Lymphangiogenesis is one of the major causes of corneal graft rejection. Among the lymphangiogenic factors, vascular endothelial growth factor (VEGF)-C and -D are considered to be the most potent. Both bind to VEGF receptor 3 (VEGFR3) to activate Prospero homeobox 1 (Prox1), a transcription factor essential for the development and maintenance of lymphatic vasculature. MicroRNAs (miRNAs) bind to the 3' untranslated regions (3' UTRs) of target genes in a sequence-specific manner and suppress gene expression. In the current study, we searched for miRNAs that target the pro-lymphangiogenic factor Prox1.

**Results:**

Among the miRNAs predicted by the bioinformatic analysis to seed match with the 3' UTR of Prox-1, we chose 3 (miR-466, miR-4305, and miR-4795-5p) for further investigation. Both the miR-466 and miR-4305 mimics, but not the miR-4795-5p mimic, significantly reduced the luciferase activity of the Prox-1 3' UTR reporter vector. In primary lymphatic endothelial cells (HDLEC), miR-466 mimic transfection suppressed Prox1 mRNA and protein expression, while miR-4305 mimic transfection did not. Experiments using mutated reporter constructs of the two possible seed match sites on the 3' UTR of Prox1 suggested that the target site 2 directly bound miR-466. HDLEC transfected with the miR-466 mimic suppressed tube formation as compared to the scrambled control. Furthermore, HDLEC transfected with a miR-466 inhibitor showed enhanced tube formation as compared to control inhibitor transfected cells, and this inhibitory effect was counteracted by Prox1 siRNA. The miR-466 mimic reduced angiogenesis and lymphangiogenesis resulting in clearer corneas in an cornea injury rat model compared to the scrambled control.

**Conclusions:**

Our data suggest that miR-446 may have a protective effect on transplanted corneas by suppressing Prox1 expression at the post-transcriptional level. The results of the current study may provide insights into the mechanisms of lymphangiogenesis resulting from corneal graft rejection and alkali-burn injuries, as well as into the development of new treatments for lymphangiogenic eye diseases.

**Electronic supplementary material:**

The online version of this article (doi:10.1186/s12929-014-0104-0) contains supplementary material, which is available to authorized users.

## Background

Approximately 10%–50% of cornea transplantation recipients experience graft rejection within one year [1]. Corneal graft rejection takes place when the immune cells of the host recognize the donor tissues as antigens and attack them. The normal cornea maintains avascularity by balancing positive and negative angiogenesis-regulating molecules such as vascular endothelial growth factor (VEGF) and angiostatin, respectively. Stimulation of cornea caused by corneal transplantation promotes the production of VEGF, disrupts the balance, and results in capillary endothelial cell proliferation and neovascularization [2,3]. Corneal lymphangiogenesis is also induced after corneal transplantation [4]. While the blood vessels provide a route of entry for CD4^+^ alloreactive T lymphocytes and memory T lymphocytes [5], newly formed corneal lymphatic vessels enable effective access of antigen presenting cells and antigenic materials to lymph nodes where accelerated sensitization to graft antigens occurs [6].

Angiogenesis, defined as the sprouting of new blood vessels from existing blood vessels, is promoted by pro-angiogenic factors, such as VEGF, angiopoietins, and integrins [7]. The potent angiogenic inducer VEGF-A can bind to both VEGF receptor 1 (VEGFR1) and VEGFR2. However, the signals responsible for inducing proliferation and migration of vascular endothelial cells are mainly transduced via VEGFR2 [8].

Lymphangiogenesis is known to be closely associated with inflammation, wound healing, corneal graft rejection, and tumors. The cell survival, proliferation, and migration of epithelial cells are important process in lymphangiogenesis, which depends on VEGF-C and -D signalling pathways through VEGFR-2 and VEGFR-3 [9,10]. Especially, VEGF-C and –D bind with VEGFR-3 and activate Prospero homeobox 1 (Prox1) [11]. Prox1 is homolog of the drosophila homeobox protein *prospero* [12]. Prox1 is a transcription factor essential for the embryonic development of vertebrates, and the development and maintenance of lymphatic vasculature in adulthood [13-15].

Following transplantation of corneas from C57BL/6 mice into BALB/C mice, the graft survival rates were compared between two experimental groups [16]. In one group, VEGF-TrapR1R2 was used to inhibit both lymphangiogenesis and angiogenesis, and in the other, VEGFR3 Ab mF4-31C was used to inhibit lymphangiogenesis only. Results showed that the survival rates of the corneal grafts were comparable in the two groups, indicating that lymphangiogenesis but not angiogenesis was an important determinant for graft survival rates.

Inflammation-induced lymphangiogenesis has been reported to be attributable to the increased expression of Prox1 stimulated by inflammatory responses [17]. In particular, Prox1 promotes the expression of the VEGF-C receptor, VEGFR3 [18]. In Prox1^+/−^ mice, milky chyle leaked from the mesenteric lymphatic vessels, and abnormal lymphatic ducts were formed [19]. Furthermore, embryos of Prox1-knockout mice showed a loss of lymphangiogenesis without disrupted hemangiogenesis from the cardinal vein [13]. Therefore, inhibiting Prox1 function or reducing Prox1 expression may be effective strategies for inhibiting corneal lymphangiogenesis.

MicroRNAs (miRNAs) are highly conserved small non-coding RNAs (19–25 nucleotides) that can modulate gene expression. Primary miRNA transcripts are processed consecutively to produce mature miRNAs by the two RNase III endonucleases, Drosha and Dicer. Mature miRNAs function as negative gene regulators through complementary sequence pairing with the 3' untranslated regions (3' UTRs) of target genes [20].

Kazenwadel et al. [21] reported that the over-expression of miR-181a in mouse lymphatic endothelial cells directly targeted the 3' UTR of Prox1, and the expression of miR-181a was lower in vascular endothelial cells than in lymphatic endothelial cells. Furthermore, the expression of miR-181a was inversely related to the expression of Prox1 [21]. Other investigators found that miR-31 targets the 3' UTR of Prox1 to suppress its expression in human lymphatic endothelial cells, and that over-expressed miR-31 led to defective lymphangiogenesis in Xenopus and zebrafish embryos [22]. However, there may be other unknown miRNAs capable of down-regulating Prox1 expression as well.

In the current study, miR-466, miR-4305, and miR-4795-5p were chosen as new miRNA candidates that could target the 3' UTR of Prox1 based on the results of a bioinformatics analysis. The ability of these miRNAs to suppress the expression of Prox1 in vitro was then investigated. The in vivo inhibitory effects of these miRNAs on lymphangiogenesis were also assessed using an experimental alkali corneal burn animal model.

## Methods

### Cells

Human dermal lymphatic endothelial cells (HDLEC) were purchased from PromoCell (Heidelberg, Germany) and cultured in MV2 media (PromoCell). HEK293T were cultured in DMEM (Gibco BRL, Grand Island, NY, USA) supplemented with 10% fetal bovine serum and antibiotics (100 U/mL penicillin and 100 μg/mL streptomycin; Gibco BRL). Both cells were incubated at 37°C and supplemented with 5% CO_2_.

### miRNA mimics, siRNA, and miRNA inhibitor

The miRNA mimics, siRNA, and scrambled miRNA used as a negative control were purchased from Genolution Pharmaceuticals (Seoul, South Korea). The sequences are as follows: scrambled control sense, 5′-UUUUAACUCAGUAUUUUUA-3′ and antisense, 5′-UAAAAAUACUGAGUUAAAA-3′; Prox1 siRNA sense, 5′- GAGUUGACAUUGGAGUGAA-3′ and antisense, 5′- UUCACUCC AAUGUCAACUC-3′. The LNA™ microRNA Power Inhibitor for hsa-miR-466 and the negative control inhibitor (NC inhibitor) were purchased from Exiqon (Vedbaek, Denmark). The sequences are as follows: inhibitor for hsa-miR-466, 5′-GTGTTGCGTGTATGTGTA-3′; NC inhibitor, 5′-GTGTAACACGTCTATACGCCCA-3′.

### Plasmid construction and site-directed mutagenesis

The full length 3' UTR of Prox1 was amplified from the genomic DNA of HEK293T cells and cloned between the Renilla luciferase coding sequence and the poly(A) site of the psiCHECK-2 plasmid (Promega, Madison, WI, USA) using XhoI/NotI sites to produce psiC-Prox1. The primers used for the amplification of 3′ UTR of Prox1 were as follows: 5′-TCGACTCGAGTGCCTACAAGAGCTGCTTCA-3′ and 5′-GGCCGCGGCCGCATTTGGCCTTTTGGGGTACT-3′. Mutations were introduced into the putative seed match sequences of psiC-Prox1 using an EZchange site-directed mutagenesis kit (Enzynomics, Daejeon, South Korea). The sequences are as follows: psiC-Prox1-m1, 5′-AATGACTTATATATGAAATCAAAATCTAGACACAT-3′ and 5′-GGGAGGCATGGATATGTTATG-3′; psiC-Prox1-m2, 5′-TTATGACTCGCCAACATTCTTTTTC-3′ and 5′-GTCTCTATTAGCAATGAAGGGAATTTGT-3′.

### Luciferase reporter assay

To test whether the miRNAs directly target the 3' UTR of Prox1, luciferase reporter assay was carried out. For this, HEK293T cells were seeded in a 96-well plate (5 × 10^3^ cells/well). After 24 h, cells were co-transfected with 20 ng psiC-Prox1 or its mutants (psiC-Prox1-m1, psiC-Prox1-m2, and psiC-Prox1-m1m2), and 10 nM each of the miRNA mimics. Luciferase activities were measured 48 h post-transfection using the Dual-Glo™ luciferase reporter assay system (Promega). Renilla luciferase activity was normalized using firefly luciferase activity for each sample.

### Transfection of HDLEC

Cells were seeded 24 h prior to transfection in 60- or 100-mm-diameter dishes containing 10 mL culture medium. Transfection was performed with 20 nM each of miRNA mimic, siRNA, and/or miRNA inhibitor using Lipofectamine™ 2000 (Invitrogen, Carlsbad, CA, USA) according to the manufacturer's protocol. Cells were harvested for RNA and protein extraction 48 h after transfection.

### Quantitative reverse transcription-polymerase chain reaction (qRT-PCR)

HDLEC were harvested and total RNA was extracted using the RNAzol™ B reagent (Tel-Test, Friendswood, TX, USA) according to the manufacturer's instruction. cDNA was synthesized using 1 μg total RNA, oligo(dT) (Macrogen, Seoul, South Korea), and M-MLV reverse transcriptase (Invitrogen). Real-time PCR for Prox1 was carried out using a SYBR green qPCR kit (Takara, Tokyo, Japan) with an Mx3000P™ Real-Time PCR System (Stratagene, La Jolla, CA, USA). The sequences of the primers were as follows: Prox1; 5′-ATCCCAGCTCCAATATGCTG-3′ and 5′-GTACTGGTGACCCCATCGTT-3′, glyceraldehyde phosphate dehydrogenase (GAPDH); 5′- ATGGGGAAGGTGAAGGTCG-3′ and 5′- GGGGTCATTGATGGCAACAATA-3′. The PCR conditions were 95°C for 10 min, followed by 40 cycles at 95°C for 20 s, 60°C for 30 s, and 72°C for 30 s. To confirm specific amplification of the PCR product, dissociation curves were checked routinely. For this, the PCR products were incubated at 95°C for 10 s and ramped up from 55°C to 95°C with a heating rate of 0.1°C/s, and fluorescence was measured continuously. Relative gene expression was calculated according to the comparative Ct method using GAPDH as an internal standard.

### Western blot analysis

To detect the Prox1 protein, cell lysate in RIPA buffer (50 μg) was mixed with NuPAGE LDS sample buffer (4×) and heated at 70°C for 10 min. The samples were electrophoretically separated on 8% SDS-PAGE gel, and then transferred to a nitrocellulose membrane (Invitrogen). The membrane was incubated overnight at 4°C with mouse monoclonal antibody against Prox1 (1:500, Abnova, Taipei City, Taiwan). After washing, the blots were incubated for 2 h at room temperature with horseradish peroxidase-conjugated anti-mouse secondary antibody (1:5000, Santa Cruz Biotechnology, Dallas, TX, USA). Protein bands were visualized using an enhanced chemiluminescence detection system (Amersham Biosciences). β-Actin antibody (Cell Signaling Technology, Danvers, MA, USA) was used to confirm comparable loading. The density of each protein band was read and quantified using Fujifilm Multi Gauge software (version 3.0).

### Tube formation assay

Endothelial cells plated on a reconstituted basement membrane matrix have been known to rapidly attach, align, and form capillary-like tubules [23]. As this endothelial cell specific process is rapid and quantifiable, tube formation assay has been used to study angiogenic and anti-angiogenic factors, to investigate mechanisms of angiogenesis, and to define endothelial cell populations [23]. To assess the effect of miRNAs on lymphangiogenesis of HDLEC, tube formation experiments were performed using MILLIPORE® In Vitro Angiogenesis Assay Kit (MILLIPORE, Billerica, MA, USA) according to the manufacturer's protocol. Ninety six-well plates were coated with cold liquid ECMatrix (70 μl/well) and incubated at 37°C in a humidified 5% CO_2_ incubator for 1 h to promote solidification. miRNA-transfected cells (7 × 10^3^cells/well) were seeded into 96-well plates pre-coated with polymerized ECMatrix and incubated with conditioned media at 37°C for 4–6 h. Formation of tube-like structures was observed under a phase-contrast microscope and quantified by counting the number of tubes formed in 3 randomly chosen fields using ImageJ software.

### Experimental corneal alkali burn animal model

Male Sprague–Dawley rats (body weight, approximately 250–300 g) were used in this study. All of the animals were treated in accordance with the guidelines of the Association for Research in Vision and Ophthalmology (ARVO) Statement for the Use of Animals in Ophthalmic and Vision Research, and the study protocol was approved by the Committee for Animal Research, Catholic University of Medicine. The rats were deeply anesthetized via intraperitoneal injection of 50 mg/kg tiletamine plus zolazepam (Zoletil; Virbac, Carros, France) and 15 mg/kg xylazine hydrochloride (Rompun; Bayer, Leuverkeusen, Germany). Alkali injuries to the eyes were induced via 10 s exposure of the central cornea to a 4-mm-diameter disk of filter paper soaked in 1 N NaOH, followed by rinsing with sterile saline (10 mL). To avoid any corneal infection, one drop of antibiotic (0.5% levofloxacin; Cravit; Santen, Osaka, Japan) was instilled onto the ocular surface immediately after the alkali burn injury. The animals were then randomly allocated to three treatment groups: scrambled control, miR-181a, and miR-466. Each group (n = 10) was treated with a single subconjunctival injection with 20 μl of 20 nM miRNA mimic immediately after the alkali burn injury.

### Immunostaining

Formalin-fixed corneas from each group of animal were embedded in paraffin and 4 μm sections were prepared for examination. To access lymphangiogenesis, corneal sections were stained with and anti-mouse lymphatic vessel endothelial hyaluronan receptor (LYVE)-1 antibody (1:500; Abcam, Eugene, OR, USA) for 16 h at 4°C. After three washes with PBS for 15 min, the sections were then stained with a Texas Red-conjugated secondary antibody (Abcam). To detect F-actin, corneal sections were incubated with rhodamine-conjugated phalloidin (dilution 1:500, Abcam) for 1 h and washed three times with PBS. The stained sections were incubated with hoechst solution to stain nucleus before examined by fluorescence microscopy at 100× magnification.

## Results

### Screening of miRNAs that can target Prox1

**Figure 1:**
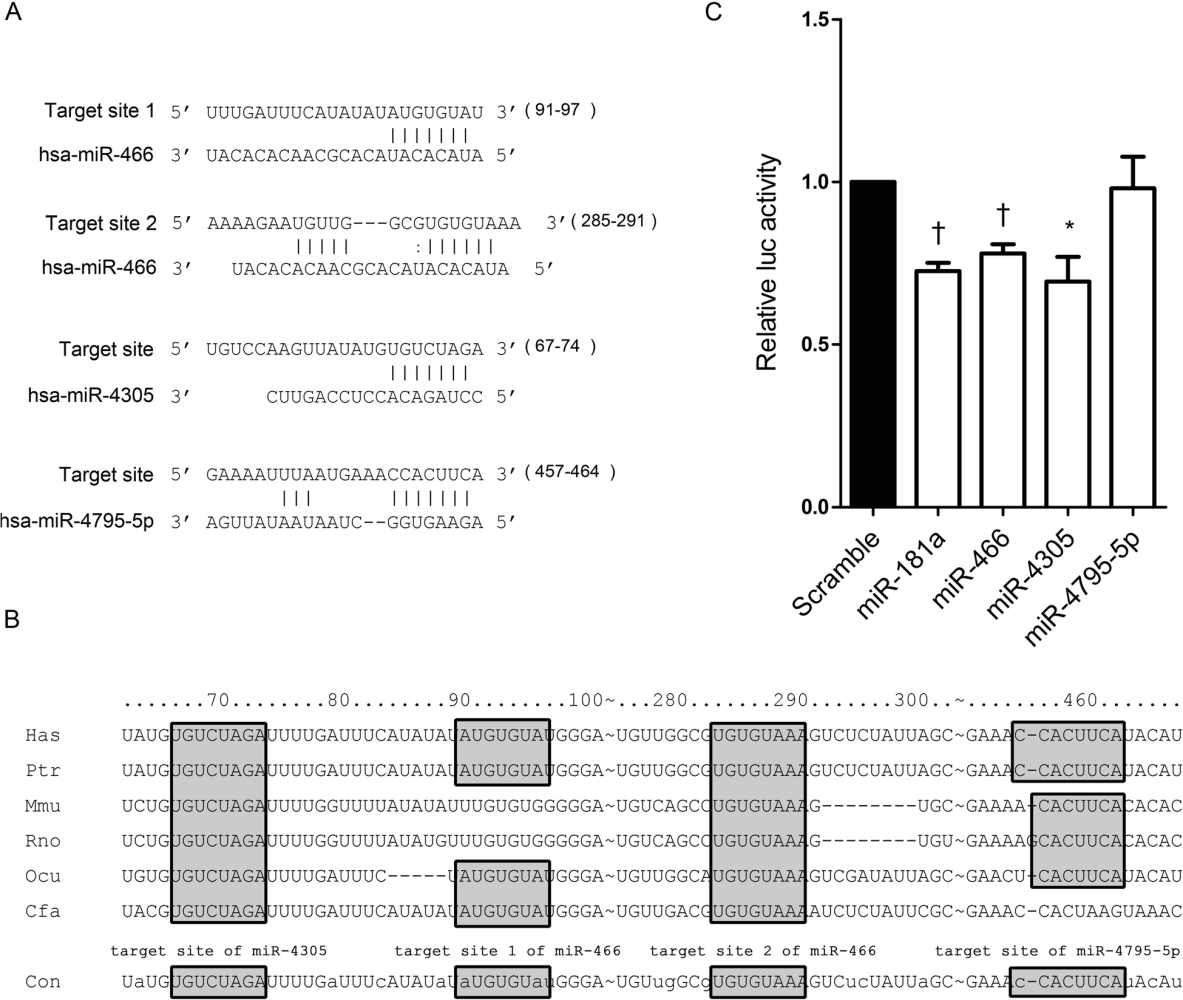
**Search for miRNAs targeting Prox1. (A)** Seed matches between the 3' UTR of Prox1 and miR-466, miR-4305, and miR-4795-5p. The coordinates for miR-466 target sites 1 and 2, as well as the target site for miR-4305 and miR-4795-5p, are shown in parentheses (GenBank accession number NM_002763). **(B)** Cross-species sequence alignment of the 3' UTR of Prox1. The shaded boxes are putative miR-466, miR-4305, and miR-4795-5p target sites. Con indicates conserved residues. Alignment of the Prox1 3' UTR shows the sequence conservation among the human (has), chimpanzee (ptr), mouse (mmu), rat (rno), rabbit (ocu), and dog (cfa). Numbers indicate the nucleotide position after the stop codon of Prox1. **(C)** Direct targeting of the Prox1 3' UTR by miR-466, miR-4305, and miR-4795-5p. Luciferase activity was measured in HEK293T cells co-transfected with psiC-Prox1 and the miR-466, miR-4305, or miR-4795-5p mimics. miR-181a, which was reported to target Prox1, was used as a positive control. Luciferase activity was normalized using internal firefly luciferase activity, and expressed as a ratio to the luciferase activity obtained from the scrambled control-transfected cells. Error bars indicate SDs (n = 3 per experiment). *P < 0.05. †P < 0.01.

In order to screen miRNAs which may target Prox1, we used publicly available TargetScan program (http://www.targetscan.org) and found 17 human microRNAs that showed a good seed match with the 3' UTR of human Prox1 mRNA (Additional file 1: Table S1). Among the 17 miRNAs, we selected miR-4305 and miR-4795-5p for further study, as they both showed 8mer seed matches with the 3' UTR of human Prox1(Figure 1A). This was considered to be important due to similarity with miR-181a, which was previously shown to target Prox1. Additionally, miR-466 was also selected, as the 3' UTR of Prox1 contained two putative binding sites for this miRNA (7mer-m8 and 7mer-1A sites), unlike other miRNAs. Although miR-4262 showed an 8mer seed match with the 3' UTR of Prox1, it was excluded from further study because the 8mer seed sequence of miR-4262 was identical to that of miR-181a. As the target sequences of miRNAs are frequently conserved in many species, we analyzed conservation of the seed match sequences in the 3' UTR of Prox1. The 7mer-1A site complementary to the target site 2 of miR-466 and the 8mer site complementary to the seed region of miR-4305 were well conserved among species (Figure 1B). However, the 7mer-m8 site complementary to the target site 1 of miR-466 and the 8mer site complementary to the seed region of miR-4795-5p were not well conserved. Subsequently, a luciferase reporter assay was conducted to assess whether miR-466, miR-4305, and miR-4795-5p directly targeted the 3' UTR of Prox1. First, HEK293T cells were co-transfected with psiC-Prox1 and each miRNA mimic. miR-181a, which is known to target Prox1, was included as a positive control, while a scrambled miRNA was used as a negative control. As expected, miR-181a significantly reduced the luciferase activity of psiC-Prox1 as compared to the scrambled control. The miR-466 and miR-4305 mimics, but not the miR-4795-5p mimic, significantly reduced the luciferase activity of the reporter vector as compared to that of the scrambled control (Figure 1C).

### Effect of miRNA mimics on the expression of Prox1 mRNA and protein

**Figure 2:**
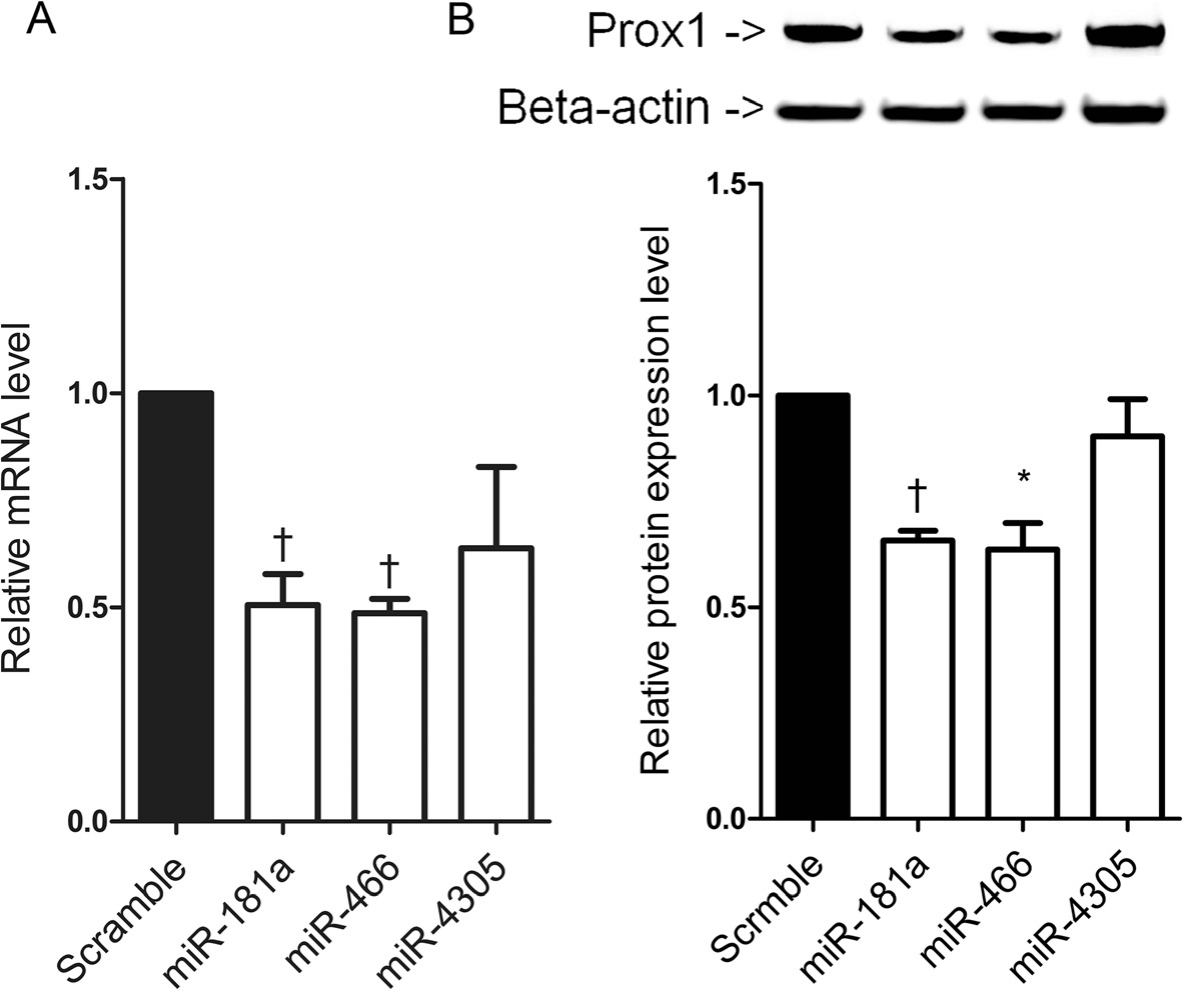
**Effects of miR-466 and miR-4305 on Prox1 expression. (A)** Reduction in Prox1 mRNA levels by miR-466. HDLEC were transfected with the miR-181a, miR-466, miR-4305 mimics, or the scrambled control. The cells were then cultured for 48 h and harvested for use in real-time qRT-PCR analyses (n = 3). Error bars indicate SDs. †P < 0.01. **(B)** Effects of miR-466 and miR-4305 on the expression of Prox1 protein. HDLEC were transfected with miR-181a, miR-466, miR-4305 mimics, or the scrambled control. The cells were cultured for 48 h and then harvested for analysis. The band densities obtained using an anti-Prox1 antibody were divided by those obtained using an anti–beta-actin antibody to normalize protein loading. Western blotting was conducted using three sets of independently transfected HDLEC. Bands were quantified using Fujifilm Multi Gauge software (version 3.0). Means were calculated using data from all three independent experiments and are expressed as ratios to the value obtained from the scrambled control-transfected HDLEC. Error bars indicate SDs. *P < 0.05. †P < 0.01.

To test whether miR-466 and miR-4305 have the ability to modulate Prox1 expression, HDLEC were harvested 48 h after miRNA mimic transfection. qRT-PCR revealed that the Prox1 mRNA level was reduced by approximately 50% following transfection with the miR-181a mimic. Similarly, the Prox1 mRNA level was decreased by about 50% following transfection with the miR-466 mimic as compared with the scrambled control (Figure 2A). miR-4305 transfection did not affect Prox1 mRNA level significantly. Western blot analysis also showed that the level of Prox1 protein was reduced by transfection with the miR-181a and miR-466 mimics as compared to levels observed following transfection with the scrambled control (Figure 2B). However, the level of Prox1 protein was not significantly affected by miR-4305.

### Dose-dependent effect of the miR-466 mimic

**Figure 3:**
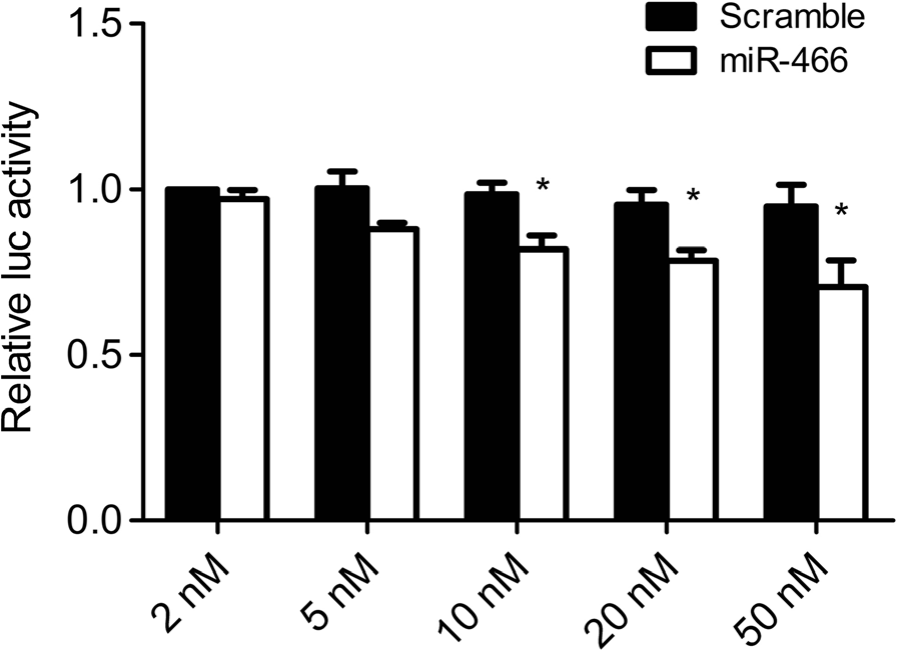
**Dose-dependent effect of the miR-466 mimic.** Luciferase activity was measured in HEK293T cells co-transfected with increasing concentrations of the miR-466 mimic and psiC-Prox1. Error bars indicate SDs (n = 3 per experiment). *P < 0.05.

As non-specific effects can obscure the results of miRNA mimic transfection experiments, we carried out a luciferase assay using increasing doses of the miR-466 mimic. To accomplish this, HEK293T cells were co-transfected with increasing concentrations of the miR-466 mimic and psiC-Prox1 reporter plasmid. Transfection with 5 nM miR-466 slightly reduced the luciferase activity, however the reduction was not statistically significant as compared to the scrambled control transfection (Figure 3). Transfecting the cells with 10 nM or higher concentrations of the miR-466 mimic caused a dose-dependent reduction in the luciferase activity of psiC-Prox1 (Figure 3). To minimize any possible non-specific effects, we used 10–20 nM miRNA mimics throughout the experiments.

### Confirming target sites for miR-466 in the Prox1 3' UTRs

**Figure 4:**
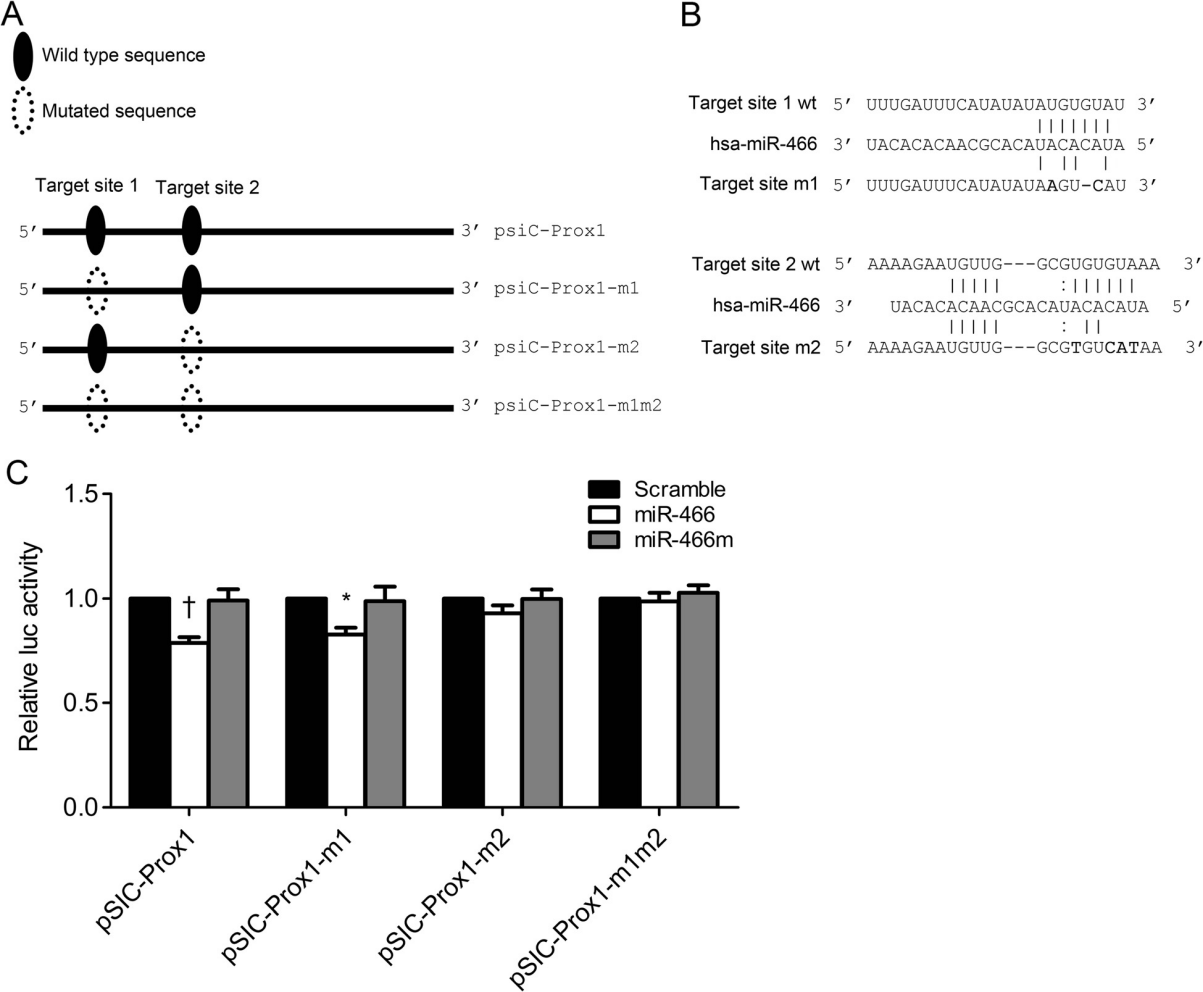
**Target site search for miR-466 in the Prox1 3' UTR. (A)** Illustration showing (i) the location of possible seed match sites between miR-466 and the 3' UTR regions and (ii) the sites altered to produce mutant forms of psiC-Prox1. Site-directed mutagenesis was performed to produce mutant versions of the 3' UTR of Prox1 seed match sequence (psiC-Prox1-m1, psiC-Prox1-m2, and psiC-Prox1-m1m2). **(B)** Seed matches between miR-466 and the mutated 3' UTRs of Prox1. wt, wild type. **(C)** Luciferase activity was measured in HEK293T cells co-transfected with the miR-466 mimic and a luciferase reporter vector containing the wild-type or mutated 3' UTRs of Prox1. The scrambled control and miR-466 m (mutant form) were used to confirm sequence-specific binding between miR-466 and the 3' UTRs. Luciferase activity was normalized using firefly luciferase activity and expressed as a ratio of the luciferase activity to the activity obtained from the scrambled control-transfected cells. Error bars indicate SDs (n = 3 per experiment). *P < 0.05. †P < 0.01.

The 3' UTR of Prox1 contains two putative binding sites for miR-466 (Figure 1A). To test whether both were directly targeted by miR-466, point mutations were introduced to psiC-Prox1 to produce psiC-Prox1-m1, psiC-Prox1-m2, and psiC-Prox1-m1m2 (Figure 4A and B). Each of these vectors was co-transfected with the miR-466 mimic into HEK293T cells, and the luciferase assay was conducted. Luciferase activity was partially reduced in the cells transfected with the miR-466 mimic together with either psiC-Prox1 or psiC-Prox1-m1 (Figure 4C). However, luciferase activity was unaffected in the cells transfected with the miR-466 mimic together with psiC-Prox1-m2 (Figure 4C). As expected from the fact that both of the putative seed match sites were eliminated, luciferase activity was not affected when psiC-Prox1-m1m2 was co-transfected with miR-466 (Figure 4C). Luciferase activity was not affected in the cells co-transfected with wild-type or mutant Prox1 3' UTR reporter vectors and miR-466 m (mutant form of miR-466) or the scrambled control (Figure 4C). These results showed that target site 2, but not the target site 1, on the 3' UTR of the Prox1 was targeted by miR-466.

### Effect of miRNAs on tube formation in HDLEC

**Figure 5:**
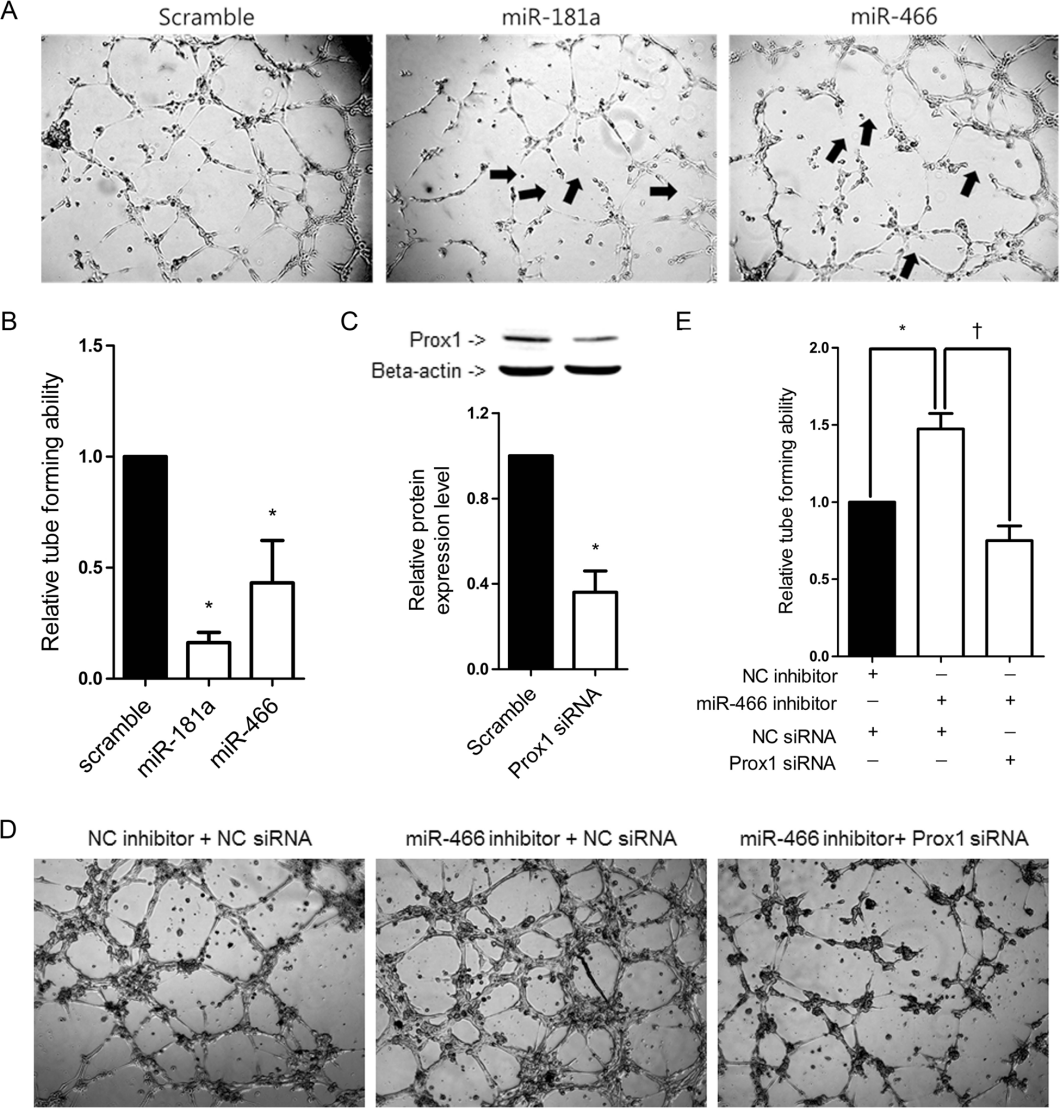
**Effect of miRNAs on tube formation in HDLEC. (A, D)** HDLEC were transfected with 20 nM each of the miRNA mimics, miRNA inhibitor and/or siRNA. After 48 h, the HDLEC cells (7 × 10^3^ cells/well) were plated on ECMatrix-coated tissue culture plates in endothelial culture medium including 10 ng/mL basic fibroblast growth factor, 5 ng/mL epidermal growth factor, 20 ng/mL insulin like growth factor 1, and 0.5 ng/mL VEGF. Capillary-like structures within the Matrigel layer were photographed after 4–6 h under a microscope (×50). Defective tube formation points are marked with black arrows. **(B)** Results similar to those in panel A were obtained in two additional independent experiments, and tube formation was quantified using the ImageJ software. Means were calculated using data from all three independent experiments and expressed as ratios to the value obtained from the scrambled control-transfected HDLEC. **(C)** Effects of Prox1 siRNA on the expression of Prox1 protein. HDLEC were transfected with 20 nM Prox1 siRNA or the scrambled control. Cells were cultured for 48 h and then harvested for analysis. Western blotting was conducted using three sets of independently transfected HDLEC. Bands were quantified using Fujifilm Multi Gauge software (version 3.0). Means were calculated using data from all three independent experiments and are expressed as ratios to the value obtained from the scrambled control-transfected HDLEC. **(E)** Results similar to those in panel D were obtained in two additional independent experiments, and tube formation was quantified using ImageJ software. Means were calculated using data from all three independent experiments and are expressed as ratios to the value obtained from the scrambled control-transfected HDLEC. Error bars indicate SDs. *P < 0.05. †P < 0.01.

To test whether miR-466 has an anti-lymphangiogenesis effect, an in vitro tube formation assay was conducted. Forty eight hours after transfection with miR-466 mimic, HDLEC were cultured on a Matrigel-coated 96 well plate for 4–6 h and the extent of tube formation was assessed. Formation of a rich network of tubular structures was observed in HDLEC transfected with the scrambled miRNA. miR-466 mimic significantly impaired the tube-forming activity of HDLEC as compared to the scrambled control (Figure 5A). Tube formation was then quantified by measuring the number of tubes formed using image manipulation software (ImageJ). The miR-181a mimic exerted the highest level of inhibitory effects on tube formation (approximately 84%) followed by the miR-466 mimic (approximately 57%; Figure 5B) when compared to the scrambled control. We also tested whether the inhibition of miR-466 enhanced tube formation in HDLEC and whether Prox1 siRNA counteracted the effect of a miR-466 inhibitor. As expected, Prox1 siRNA transfection significantly decreased Prox1 expression in HDLEC as compared to transfection with the scrambled control (Figure 5C). HDLEC were then transfected with a miR-466 inhibitor alone or together with Prox1 siRNA. HDLEC transfected with the miR-466 inhibitor alone showed significantly increased tube formation (Figure 5D and E). However, co-transfected Prox1 siRNA counteracted the effect of the miR-466 inhibitor (Figure 5D and E).

### Effect of miRNAs on angiogenesis and lymphangiogenesis in the corneal alkali burn animal model

**Figure 6:**
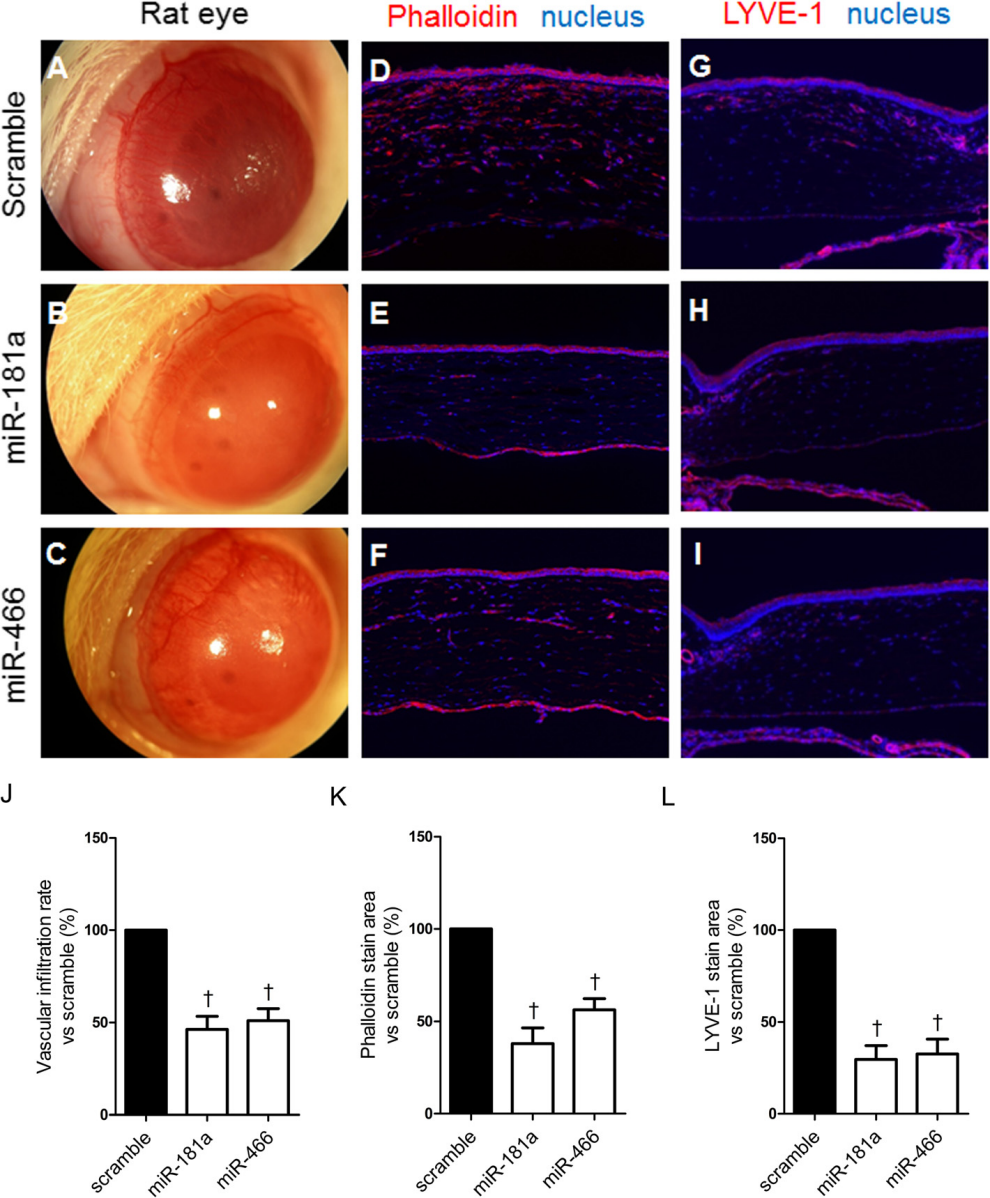
**Inhibition of corneal neovascularization by miRNAs in a corneal injury animal model.** Alkali burn-induced corneas of Sprague–Dawley rats were treated once with each miRNA mimic by subconjunctival injection on the day of injury. **(A-C)** Photographic images of corneas treated with each miRNA mimic. Immunofluorescent stain of corneal sections to detect vessel formation **(D-F)** or lymphangiogenesis **(G-I)**. Red stains F-actin **(D-F)** or LYVE-1 **(G-I)**, which is a marker of vessels and lymphatic vessels, and blue shows hoechst staining **(D-I)**. Photographs of corneas were taken two weeks after the treatment. **(J)** Quantification of new blood vessel formation, F-actin-stained area **(K)**, and LYVE-1-stained area **(L)**. Relative rate was quantified using ImageJ software and normalized to the value obtained from the scrambled control-injected rats. Error bars indicate SDs. †P < 0.01.

The effects of miRNAs on corneal opacity, angiogenesis, and lymphangiogenesis were examined in an experimental animal model. Immediately after inducing an alkali burn, each miRNA mimic was subconjunctivally injected once, and the effect of each miRNA was evaluated two weeks later. miR-181a was used as a positive control, while the scrambled miRNA was used as a negative control. In the scrambled control-injected animals, the injured central corneal stroma appeared opaque with a distinct edematous margin (Figure 6A). In contrast, miR-466- or miR-181a-injected animals showed reduced opacity (Figure 6B and 6C). Under direct microscopic observation, blood vascular infiltration was about 46% of the scrambled control group level in the miR-181a-treated group. When the animals were injected with miR-466, blood vascular infiltration was 51% of the scrambled control group level (Figure 6J). Infiltration of blood vessel into the cornea was examined by staining with phalloidin to detect F-actin (Figure 6D-F), and positive cells were quantified using the ImageJ software. The cornea section from the scrambled miRNA-injected eyes showed a thickened cornea and strong scattered F-actin staining. In the miR-181a-treated group, the cornea showed almost half the thickness and F-actin positive staining was about 38% compared to the scrambled control group (Figure 6K). When the animals were injected with miR-466, corneal thickness was between those observed for the scrambled control and the miR-181a-injected animals, while F-actin positive staining was 56% of the scrambled control group level (Figure 6K). To analyze the effects of miRNAs on lymphangiogenesis of the cornea, corneal sections from each animal group were analyzed by immunohistofluorescence staining with anti-LYVE-1 antibodies (Figure 6G-I). miR-466- and miR-181a-injected corneas showed significantly reduced levels of LYVE-1 staining (~33% and ~30%, respectively) compared to the scrambled control-treated corneas (Figure 6L).

## Discussion

The results of the current study showed that the expression of Prox1 was inhibited by miR-466 at both the mRNA and protein levels. The luciferase assay showed that miR-466 directly targeted the well conserved 7mer-1A site in the 3' UTR of Prox1 (target site 2), as the suppressive effect of the miR-466 mimic on luciferase activity was almost abolished when this site was mutated. The target site 2, unlike the target site 1, was well conserved among species. Previous reports showed that additional Watson-Crick pairing to four or five sequential nucleotides at nucleotides 12–17 enhanced miRNA targeting [24]. The target site 2 contains five contiguous sequences that can be used for an effective 3' pairing with the nucleotides 14 ~ 18 of miR-466, while the target site 1 does not. Thus, the target site 2 seems to have better chance to be targeted with miR-466 than the target site 1.

In the alkali burn corneal injury model, miR-466 decreased corneal opacity and inhibited both lymphangiogenesis and angiogenesis, possibly attributable to decreased Prox1 expression. According to the results of a recent study [25], growth and migration of vascular endothelial cells were decreased by the addition of culture supernatant derived from Prox1 siRNA-treated oral squamous cell carcinoma cell cultures. In that experiment, down-regulation of VEGF-C was observed following silencing of the Prox1 gene [25]. These results suggested that Prox1 acted as a regulator of angiogenesis and lymphangiogenesis in oral squamous cell carcinoma [25]. Prox1 is known to control the expression of angiopoietin-2, which promotes angiogenesis in vascular endothelial cells [26]. VEGF-C and angiopoietin-2 are also important modulators of angiogenesis [27,28]. Therefore, suppressed Prox1 expression by miR-466 may have inhibited the expression of angiogenic modulators such as VEGF-C and angiopoietin-2, resulting in reduced angiogenesis in the alkali burn corneal injury animal model. However, as miRNAs usually have multiple targets, our observations may be attributable to angiogenic modulators other than Prox1 targeted by miR-466. This notion is supported by previous findings demonstrating that miR-466 induced apoptosis by targeting a few anti-apoptotic genes [29], and that this induced apoptosis resulted in the inhibition of angiogenesis [30,31].

mmu-miR-466, which can be induced by apoptosis [29] and metabolic oxidative stress [32], was shown to enhance viral replication by inhibition of INF-α [33]. In addition, over-expression of mmu-miR-466 inhibited Nfat5 expression and was associated with renal dysfunction [34]. However, the functions of hsa-miR-466 in human cells have yet to be fully elucidated. The results of the current study demonstrated that hsa-miR-466 inhibited Prox1 expression and suppressed tube formation in human primary lymphatic endothelial cells. Furthermore, miR-466 reduced angiogenesis and lymphangiogenesis, resulting in clearer corneas than those observed in the scrambled control-treated mice in an animal cornea injury model.

The inhibitory effects of miR-466 on Prox1 expression, tube formation, and lymphatic vessel formation were comparable to those of miR-181. However, the inhibitory effect of miR-466 on blood vessel formation in the in vivo corneal injury model was slightly weaker than that of miR-181a. Findings of discrepant efficiencies attributed to miRNAs measured using different methods are not rare [35,36] and may be due to different experimental settings, or may reflect experimental errors. Another possibility is that miR-181a may also target angiogenesis-related genes other than Prox1 more effectively than miR-466.

miR-4305 also significantly reduced the luciferase activity of the Prox1 3' UTR reporter vector as compared to the scrambled control in this study. However, Prox1 expression was not changed following miR-4305 mimic transfection of HDLEC compared to the scrambled control transfection. It is not clear why the miR-4305 mimic showed discrepant results in the luciferase assay, and in qRT-PCR and western blot experiments. These discrepant findings may be due to the use of only the 3' UTR of Prox1, as opposed to the whole Prox1 mRNA, in the luciferase assay. The seed match sequence on the 3' UTR of Prox1 in the luciferase reporter construct may have been available for miR-4305 binding, while the confirmation of that sequence in the Prox1 mRNA may have not allowed for miR-4305 binding. In addition, HEK293T cells were used for the luciferase assay, while HDLEC were used for other experiments.

Bevacizumab is a recombinant humanized immunoglobulin G1 monoclonal antibody against all isoforms of VEGF-A [37]. Bevacizumab binds VEGF-A and prevents the interaction of VEGF-A to its receptor on the surface of endothelial cells, leading to inhibition of endothelial cell proliferation and new blood vessel formation [38]. Since the FDA approved bevacizumab for the treatment of metastatic colorectal cancer in combination with 5-fluorouracil, bevacizumab has been used for various malignancies [38,39]. Although bevacizumab-based treatment on eye diseases has not been approved by the FDA, intravitreal bevacizumab injection led to improvement of visual acuity and regression of retinal neovascularization in proliferative diabetic retinopathy patients [40]. Likewise, intraocular pressure and angiogenesis decreased following the administration of bevacizumab eye drops in neovascular glaucoma patients [41]. Furthermore, subconjunctival administration of bevacizumab after corneal transplantation decreased the number and caliber of vessels, however the effects were transient [42]. Bevacizumab inhibits only VEGF-A-induced lymphangiogenesis and has no effects on the lymphangiogenesis caused by VEGF-C or VEGF-D [43]. To treat lymphangiogenesis-related disease more effectively, it is necessary to develop drugs that directly target the process of lymphangiogenesis.

The results of the current study demonstrated that miR-466 inhibited Prox1, which is known to be activated by various growth factors including VEGF-A, −C, and -D [11,44]. Thus, miR-466 is expected to have broader and greater inhibitory effects on lymphangiogenesis-related diseases than bevacizumab.

## Conclusions

Our results on human lymphatic endothelial cell and alkali burn corneal injury model demonstrated that miR-466 directly targeted the 3' UTR of Prox1 and suppressed the expression of Prox1, resulting in inhibition of lymphangiogenesis. Therefore, miR-466 may be useful for investigating the mechanisms of lymphangiogenesis and for developing treatments for lymphangiogenic eye diseases.

## Additional file

Additional file 1: TableS1. Predicted miRNAs on target Prox1 gene(TargetScan 6.2, http://www.targetscan.org/).

## Abbreviations

VEGF: Vascular endothelial growth factor
VEGFR: VEGF receptor
Prox1: Prospero homeobox 1
miRNA: MicroRNA
3' UTR: 3' untranslated regions
HDLEC: Human primary lymphatic endothelial cell
GAPDH: Glyceraldehyde phosphate dehydrogenase
LYVE: Lymphatic vessel endothelial hyaluronan receptor
qRT-PCR: Quantitative reverse transcription-polymerase chain reaction
