# Supplementary material for: MicroRNA miR-466 inhibits Lymphangiogenesis by targeting prospero-related homeobox 1 in the alkali burn corneal injury model
Source: J Biomed Sci. 2015 Jan 2;22(1):3. doi: 10.1186/s12929-014-0104-0 (PMC4304626; doi:10.1186/s12929-014-0104-0)
Supplement: Additional file 1: TableS1. — Predicted miRNAs on target Prox1 gene(TargetScan 6.2, http://www.targetscan.org/). [file 12929_2014_104_MOESM1_ESM.pdf]

Supplementary Table 1 | Predicted miRNAs on target Prox1 gene (TargetScan 6.2, http://www.targetscan.org/.)

microRNA	Seed match	Selected microRNA
hsa-miR-181	8mer	(already known)
hsa-miR-4262	8mer	(identical seed with miR-181)
hsa-miR-4305	8mer	*
hsa-miR-4795-5p	8mer	*
hsa-miR-466	7mer-m8<br>7mer-1A	*
hsa-miR-573	7mer-m8	
hsa-miR-592	7mer-m8	
hsa-miR-635	7mer-m8	
hsa-miR-3189-3p	7mer-m8	
hsa-miR-3616-5p	7mer-m8	
hsa-miR-3647-5p	7mer-m8	
hsa-miR-4509	7mer-m8	
hsa-miR-4512	7mer-m8	
hsa-miR-4520b-3p	7mer-m8	
hsa-miR-597	7mer-1A	
hsa-miR-4520a-3p	7mer-1A	
hsa-miR-4672	7mer-1A	
